# *catena*-Poly[[diazido­zinc(II)]-μ-di-4-pyridylamine-κ^2^ *N*:*N*′]

**DOI:** 10.1107/S1600536809051599

**Published:** 2009-12-04

**Authors:** Aaron M. Hardy, Robert L. LaDuca

**Affiliations:** a Lyman Briggs College, Department of Chemistry, Michigan State University, East Lansing, MI 48825 USA

## Abstract

In the title compound, [Zn(N_3_)_2_(C_10_H_9_N_3_)]_*n*_, tetra­hedrally coordinated Zn^II^ ions with two monodentate azide ligands are linked into zigzag one-dimensional chain motifs by di-4-pyridylamine (dpa) tethers. Individual [Zn(N_3_)_2_(dpa)]_*n*_ chains are connected into supra­molecular layers *via* N—H⋯N hydrogen bonding between the central amine groups of the dpa ligands and terminal unligated azide N atoms. The azide ligands in one supra­molecular layer penetrate through the neighboring layers above and below, allowing stacking into a three-dimensional structure.

## Related literature

For other coordination polymers containing dpa ligands, see: LaDuca (2009 ▶). For the preparation of dpa, see: Zapf *et al.* (1998 ▶).
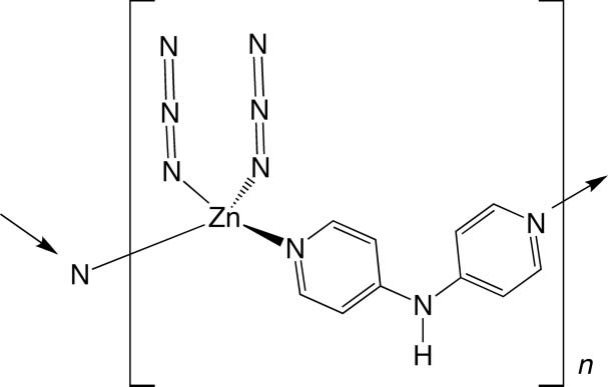


## Experimental

### 

#### Crystal data

[Zn(N_3_)_2_(C_10_H_9_N_3_)]
*M* *_r_* = 320.63
Monoclinic, 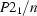

*a* = 6.7988 (2) Å
*b* = 16.0105 (5) Å
*c* = 11.7733 (4) Å
β = 99.904 (1)°
*V* = 1262.45 (7) Å^3^
*Z* = 4
Mo *K*α radiation
μ = 1.95 mm^−1^
*T* = 173 K
0.40 × 0.30 × 0.20 mm

#### Data collection

Bruker APEXII diffractometer
Absorption correction: multi-scan (*SADABS*; Sheldrick, 1996 ▶) *T*_min_ = 0.628, *T*_max_ = 0.745
11312 measured reflections
2306 independent reflections
2186 reflections with *I* > 2σ(*I*)
*R*_int_ = 0.021

#### Refinement

*R*[*F* ^2^ > 2σ(*F* ^2^)] = 0.020
*wR*(*F* ^2^) = 0.056
*S* = 1.11
2306 reflections
184 parameters
H atoms treated by a mixture of independent and constrained refinement
Δρ_max_ = 0.28 e Å^−3^
Δρ_min_ = −0.30 e Å^−3^

### 

Data collection: *APEX2* (Bruker, 2006 ▶); cell refinement: *SAINT* (Bruker, 2006 ▶); data reduction: *SAINT*; program(s) used to solve structure: *SHELXS97* (Sheldrick, 2008 ▶); program(s) used to refine structure: *SHELXL97* (Sheldrick, 2008 ▶); molecular graphics: *CrystalMaker* (Palmer, 2007 ▶); software used to prepare material for publication: *SHELXL97*.

## Supplementary Material

Crystal structure: contains datablocks I, global. DOI: 10.1107/S1600536809051599/zl2257sup1.cif

Structure factors: contains datablocks I. DOI: 10.1107/S1600536809051599/zl2257Isup2.hkl

Additional supplementary materials:  crystallographic information; 3D view; checkCIF report

## Figures and Tables

**Table 1:** Hydrogen-bond geometry (Å, °)

*D*—H⋯*A*	*D*—H	H⋯*A*	*D*⋯*A*	*D*—H⋯*A*
N8—H8*N*⋯N3^i^	0.81 (2)	2.14 (2)	2.938 (2)	172.7 (19)

Symmetry code: (i) 
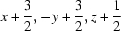
.

**Fig. 1.**
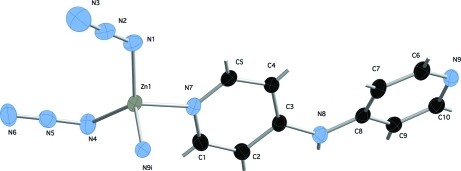
The coordination environment of the title compound, showing 50% probability ellipsoids and the atom numbering scheme. Hydrogen atom positions are shown as grey sticks. Color codes: grey Zn, light blue N, black C. Symmetry code: (i) -x + 3/2, y -1/2, -z + 3/2.

**Fig. 2.**
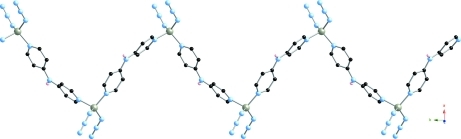
A single [Zn(N3)2(dpa)]n chain.

**Fig. 3.**
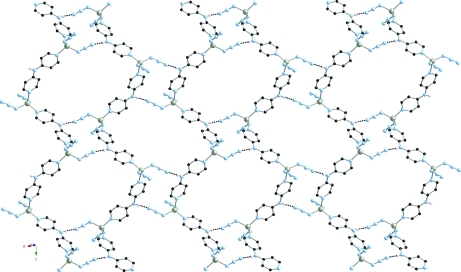
Supramolecular layer of [Zn(N3)2(dpa)]n chains. N—H···N hydrogen bonding is shown as dashed lines.

**Fig. 4.**
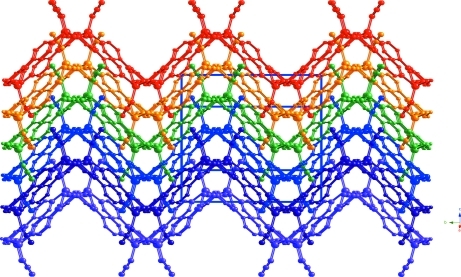
Stacking of supramolecular layers in the title compound.

## supplementary crystallographic information

### Comment

In recent years we have been exploring the use of di-4-pyridylamine (dpa) as a neutral dipodal tethering ligand for the construction of divalent metal coordination polymers (LaDuca, 2009). This chemistry was extended into a system with azido ligands, with the synthesis and characterization of a divalent zinc coordination polymer, [Zn(N_3_)_2_(dpa)]_n_.

The asymmetric unit of the title compound contains a Zn^II^ ion, two azido ligands, and one dpa moiety (Fig. 1). Distorted tetrahedral [ZnN_4_] coordination is observed, with two N donors from two monodentate azido ligands and two pyridyl N donors from two different dpa ligands. The dpa ligands link the Zn^II^ ions into zigzag [Zn(N_3_)_2_(dpa)]_n_ one-dimensional coordination polymer chains (Fig. 2), which are oriented parallel to the *b* crystal direction.

Individual [Zn(N_3_)_2_(dpa)]_n_ chains are connected into supramolecular layers *via* N—H···N hydrogen bonding between the central amine groups of the dpa ligands and terminal unligated azide N atoms (Fig. 3). These layers stack to form the three-dimensional crystal structure of the title compound, with their pendant azido ligands penetrating through the layer above and the layer below (Fig. 4).

### Experimental

All starting materials were obtained commercially, except for dpa, which was prepared by a published procedure (Zapf *et al.*, 1998). Zinc nitrate hexahydrate (30 mg, 0.10 mmol) was dissolved in 3 mL H_2_O in a glass vial. A solution of sodium azide (13 mg, 0.20 mmol) in 1.5 mL tetrahydrofuran was carefully layered on top of the aqueous solution, followed by a solution of dpa (17 mg, 0.10 mmol) in 1.5 mL methanol. The reaction mixture was allowed to stand undisturbed at 293 K for 14 days, whereupon colourless crystals of the title compound (23 mg, 72% yield) had precipitated.

### Refinement

All H atoms bound to C atoms were placed in calculated positions, with C—H = 0.95 Å, and refined in riding mode with *U*_iso_ = 1.2*U*_eq_(C). The H atom bound to the dpa amine N atom was found in a difference Fourier map, and refined with *U*_iso_ = 1.2*U*_eq_(N).

### Crystal data

**Table:**

[Zn(N_3_)_2_(C_10_H_9_N_3_)]	*F*(000) = 648
*M**_r_* = 320.63	*D*_x_ = 1.687 Mg m^−^^3^
Monoclinic, *P*2_1_/*n*	Mo *K*α radiation, λ = 0.71073 Å
Hall symbol: -P 2yn	Cell parameters from 11312 reflections
*a* = 6.7988 (2) Å	θ = 2.2–25.4°
*b* = 16.0105 (5) Å	µ = 1.95 mm^−^^1^
*c* = 11.7733 (4) Å	*T* = 173 K
β = 99.904 (1)°	Block, colourless
*V* = 1262.45 (7) Å^3^	0.40 × 0.30 × 0.20 mm
*Z* = 4	

### Data collection

**Table:**

Bruker APEXII diffractometer	2306 independent reflections
Radiation source: fine-focus sealed tube	2186 reflections with *I* > 2σ(*I*)
graphite	*R*_int_ = 0.021
ω/φ scans	θ_max_ = 25.4°, θ_min_ = 2.2°
Absorption correction: multi-scan (*SADABS*; Sheldrick, 1996)	*h* = −7→8
*T*_min_ = 0.628, *T*_max_ = 0.745	*k* = −19→19
11312 measured reflections	*l* = −12→14

### Refinement

**Table:**

Refinement on *F*^2^	Primary atom site location: structure-invariant direct methods
Least-squares matrix: full	Secondary atom site location: difference Fourier map
*R*[*F*^2^ > 2σ(*F*^2^)] = 0.020	Hydrogen site location: inferred from neighbouring sites
*wR*(*F*^2^) = 0.056	H atoms treated by a mixture of independent and constrained refinement
*S* = 1.11	*w* = 1/[σ^2^(*F*_o_^2^) + (0.0314*P*)^2^ + 0.4475*P*] where *P* = (*F*_o_^2^ + 2*F*_c_^2^)/3
2306 reflections	(Δ/σ)_max_ = 0.001
184 parameters	Δρ_max_ = 0.28 e Å^−^^3^
0 restraints	Δρ_min_ = −0.30 e Å^−^^3^

### Special details

**Table:**

Geometry. All e.s.d.'s (except the e.s.d. in the dihedral angle between two l.s. planes) are estimated using the full covariance matrix. The cell e.s.d.'s are taken into account individually in the estimation of e.s.d.'s in distances, angles and torsion angles; correlations between e.s.d.'s in cell parameters are only used when they are defined by crystal symmetry. An approximate (isotropic) treatment of cell e.s.d.'s is used for estimating e.s.d.'s involving l.s. planes.
Refinement. Refinement of *F*^2^ against ALL reflections. The weighted *R*-factor *wR* and goodness of fit *S* are based on *F*^2^, conventional *R*-factors *R* are based on *F*, with *F* set to zero for negative *F*^2^. The threshold expression of *F*^2^ > σ(*F*^2^) is used only for calculating *R*-factors(gt) *etc*. and is not relevant to the choice of reflections for refinement. *R*-factors based on *F*^2^ are statistically about twice as large as those based on *F*, and *R*- factors based on ALL data will be even larger.

### Fractional atomic coordinates and isotropic or equivalent isotropic displacement parameters (Å^2^)

**Table:**

	*x*	*y*	*z*	*U*_iso_*/*U*_eq_	
Zn1	0.18762 (2)	0.669988 (11)	0.844834 (15)	0.02285 (8)	
N1	−0.0205 (2)	0.72140 (9)	0.72917 (12)	0.0298 (3)	
N2	−0.1690 (2)	0.68678 (9)	0.68471 (13)	0.0300 (3)	
N3	−0.3142 (3)	0.65531 (12)	0.64086 (16)	0.0514 (5)	
N4	0.1421 (2)	0.63906 (9)	0.99828 (12)	0.0313 (3)	
N5	−0.0053 (2)	0.60474 (8)	1.01875 (11)	0.0282 (3)	
N6	−0.1423 (2)	0.57318 (10)	1.04586 (15)	0.0408 (4)	
N7	0.41836 (19)	0.75077 (8)	0.87138 (11)	0.0243 (3)	
N8	0.9592 (2)	0.88055 (8)	0.91029 (12)	0.0250 (3)	
H8N	1.029 (3)	0.8687 (12)	0.9705 (17)	0.030*	
N9	1.20751 (19)	1.06963 (8)	0.73394 (11)	0.0244 (3)	
C1	0.5713 (2)	0.73558 (10)	0.95831 (14)	0.0269 (3)	
H1	0.5554	0.6945	1.0119	0.032*	
C2	0.7489 (2)	0.77786 (10)	0.97125 (14)	0.0261 (3)	
H2	0.8507	0.7651	1.0322	0.031*	
C3	0.7764 (2)	0.84063 (9)	0.89209 (14)	0.0225 (3)	
C4	0.6151 (2)	0.85951 (10)	0.80644 (14)	0.0258 (3)	
H4	0.6232	0.9030	0.7551	0.031*	
C5	0.4424 (2)	0.81284 (10)	0.79843 (14)	0.0250 (3)	
H5	0.3371	0.8251	0.7393	0.030*	
C6	1.0598 (2)	1.02151 (10)	0.67845 (14)	0.0273 (3)	
H6	1.0141	1.0322	0.6007	0.033*	
C7	0.9715 (2)	0.95715 (10)	0.72946 (14)	0.0269 (3)	
H7	0.8711	0.9251	0.6867	0.032*	
C8	1.0360 (2)	0.94115 (9)	0.84654 (13)	0.0229 (3)	
C9	1.1960 (2)	0.98884 (10)	0.90319 (14)	0.0245 (3)	
H9	1.2484	0.9782	0.9801	0.029*	
C10	1.2754 (2)	1.05127 (10)	0.84526 (14)	0.0252 (3)	
H10	1.3811	1.0823	0.8848	0.030*	

### Atomic displacement parameters (Å^2^)

**Table:**

	*U*^11^	*U*^22^	*U*^33^	*U*^12^	*U*^13^	*U*^23^
Zn1	0.01842 (12)	0.02553 (12)	0.02505 (12)	−0.00136 (6)	0.00501 (8)	−0.00143 (6)
N1	0.0248 (7)	0.0320 (7)	0.0307 (7)	0.0018 (6)	−0.0005 (6)	−0.0007 (6)
N2	0.0294 (8)	0.0338 (7)	0.0259 (7)	0.0060 (6)	0.0018 (6)	−0.0030 (6)
N3	0.0444 (11)	0.0571 (11)	0.0448 (10)	−0.0097 (8)	−0.0145 (8)	−0.0019 (8)
N4	0.0296 (7)	0.0384 (8)	0.0265 (7)	−0.0070 (6)	0.0060 (6)	0.0012 (6)
N5	0.0320 (8)	0.0277 (7)	0.0255 (7)	0.0014 (6)	0.0065 (6)	0.0009 (6)
N6	0.0387 (9)	0.0395 (9)	0.0483 (9)	−0.0061 (7)	0.0193 (7)	0.0040 (7)
N7	0.0202 (6)	0.0268 (7)	0.0266 (7)	−0.0021 (5)	0.0059 (5)	−0.0020 (5)
N8	0.0218 (7)	0.0269 (7)	0.0256 (7)	−0.0035 (5)	0.0020 (5)	0.0013 (5)
N9	0.0226 (6)	0.0252 (6)	0.0264 (7)	−0.0006 (5)	0.0073 (5)	−0.0012 (5)
C1	0.0272 (8)	0.0271 (8)	0.0266 (8)	−0.0026 (6)	0.0048 (6)	0.0027 (6)
C2	0.0236 (8)	0.0274 (8)	0.0260 (8)	−0.0010 (6)	0.0007 (6)	0.0020 (6)
C3	0.0212 (8)	0.0220 (7)	0.0253 (8)	0.0001 (6)	0.0070 (6)	−0.0044 (6)
C4	0.0239 (8)	0.0256 (8)	0.0285 (8)	−0.0004 (6)	0.0067 (6)	0.0041 (6)
C5	0.0200 (8)	0.0290 (8)	0.0257 (8)	0.0009 (6)	0.0032 (6)	−0.0003 (6)
C6	0.0257 (8)	0.0340 (9)	0.0230 (8)	−0.0024 (7)	0.0067 (6)	−0.0029 (6)
C7	0.0239 (8)	0.0317 (8)	0.0257 (8)	−0.0058 (6)	0.0061 (6)	−0.0069 (6)
C8	0.0203 (7)	0.0219 (7)	0.0279 (8)	0.0014 (6)	0.0079 (6)	−0.0028 (6)
C9	0.0218 (7)	0.0256 (8)	0.0256 (8)	0.0001 (6)	0.0029 (6)	0.0003 (6)
C10	0.0214 (8)	0.0251 (8)	0.0288 (8)	−0.0010 (6)	0.0032 (6)	−0.0025 (6)

### Geometric parameters (Å, °)

**Table:**

Zn1—N4	1.9486 (14)	C1—H1	0.9300
Zn1—N1	1.9676 (14)	C2—C3	1.405 (2)
Zn1—N7	2.0157 (13)	C2—H2	0.9300
Zn1—N9^i^	2.0439 (13)	C3—C4	1.390 (2)
N1—N2	1.191 (2)	C4—C5	1.381 (2)
N2—N3	1.149 (2)	C4—H4	0.9300
N4—N5	1.203 (2)	C5—H5	0.9300
N5—N6	1.152 (2)	C6—C7	1.381 (2)
N7—C5	1.342 (2)	C6—H6	0.9300
N7—C1	1.350 (2)	C7—C8	1.396 (2)
N8—C3	1.381 (2)	C7—H7	0.9300
N8—C8	1.383 (2)	C8—C9	1.400 (2)
N8—H8N	0.81 (2)	C9—C10	1.372 (2)
N9—C6	1.343 (2)	C9—H9	0.9300
N9—C10	1.345 (2)	C10—H10	0.9300
C1—C2	1.370 (2)		
			
N4—Zn1—N1	122.54 (6)	N8—C3—C4	126.08 (15)
N4—Zn1—N7	105.23 (6)	N8—C3—C2	116.56 (14)
N1—Zn1—N7	106.66 (6)	C4—C3—C2	117.28 (14)
N4—Zn1—N9^i^	110.15 (6)	C5—C4—C3	119.17 (15)
N1—Zn1—N9^i^	106.28 (6)	C5—C4—H4	120.4
N7—Zn1—N9^i^	104.59 (5)	C3—C4—H4	120.4
N2—N1—Zn1	124.32 (12)	N7—C5—C4	123.57 (15)
N3—N2—N1	178.26 (19)	N7—C5—H5	118.2
N5—N4—Zn1	124.92 (12)	C4—C5—H5	118.2
N6—N5—N4	175.50 (17)	N9—C6—C7	124.14 (15)
C5—N7—C1	117.13 (13)	N9—C6—H6	117.9
C5—N7—Zn1	123.60 (11)	C7—C6—H6	117.9
C1—N7—Zn1	118.65 (10)	C6—C7—C8	118.68 (15)
C3—N8—C8	130.87 (14)	C6—C7—H7	120.7
C3—N8—H8N	113.9 (14)	C8—C7—H7	120.7
C8—N8—H8N	115.0 (14)	N8—C8—C7	125.49 (14)
C6—N9—C10	116.84 (14)	N8—C8—C9	117.32 (14)
C6—N9—Zn1^ii^	121.35 (11)	C7—C8—C9	117.16 (14)
C10—N9—Zn1^ii^	121.73 (11)	C10—C9—C8	120.02 (15)
N7—C1—C2	123.00 (15)	C10—C9—H9	120.0
N7—C1—H1	118.5	C8—C9—H9	120.0
C2—C1—H1	118.5	N9—C10—C9	123.03 (14)
C1—C2—C3	119.69 (15)	N9—C10—H10	118.5
C1—C2—H2	120.2	C9—C10—H10	118.5
C3—C2—H2	120.2		
			
N4—Zn1—N1—N2	−67.03 (16)	C1—C2—C3—C4	3.1 (2)
N7—Zn1—N1—N2	171.88 (14)	N8—C3—C4—C5	179.10 (15)
N9^i^—Zn1—N1—N2	60.70 (15)	C2—C3—C4—C5	−4.2 (2)
N1—Zn1—N4—N5	43.33 (17)	C1—N7—C5—C4	1.7 (2)
N7—Zn1—N4—N5	165.09 (14)	Zn1—N7—C5—C4	−169.13 (12)
N9^i^—Zn1—N4—N5	−82.70 (15)	C3—C4—C5—N7	1.9 (2)
N4—Zn1—N7—C5	−149.52 (12)	C10—N9—C6—C7	2.1 (2)
N1—Zn1—N7—C5	−17.95 (14)	Zn1^ii^—N9—C6—C7	−174.54 (12)
N9^i^—Zn1—N7—C5	94.40 (13)	N9—C6—C7—C8	1.1 (2)
N4—Zn1—N7—C1	39.83 (13)	C3—N8—C8—C7	−22.3 (3)
N1—Zn1—N7—C1	171.39 (11)	C3—N8—C8—C9	159.76 (15)
N9^i^—Zn1—N7—C1	−76.26 (12)	C6—C7—C8—N8	178.29 (15)
C5—N7—C1—C2	−2.9 (2)	C6—C7—C8—C9	−3.8 (2)
Zn1—N7—C1—C2	168.40 (13)	N8—C8—C9—C10	−178.40 (14)
N7—C1—C2—C3	0.5 (2)	C7—C8—C9—C10	3.5 (2)
C8—N8—C3—C4	−6.7 (3)	C6—N9—C10—C9	−2.4 (2)
C8—N8—C3—C2	176.62 (15)	Zn1^ii^—N9—C10—C9	174.18 (12)
C1—C2—C3—N8	−179.89 (14)	C8—C9—C10—N9	−0.4 (2)

Symmetry codes: (i) −*x*+3/2, *y*−1/2, −*z*+3/2; (ii) −*x*+3/2, *y*+1/2, −*z*+3/2.

### Hydrogen-bond geometry (Å, °)

**Table:**

*D*—H···*A*	*D*—H	H···*A*	*D*···*A*	*D*—H···*A*
N8—H8N···N3^iii^	0.81 (2)	2.14 (2)	2.938 (2)	172.7 (19)

Symmetry codes: (iii) *x*+3/2, −*y*+3/2, *z*+1/2.
